# The Mediating Effect of Marital Intimacy on the Relationship between Spouse-Related Stress and Prenatal Depression in Pregnant Couples: An Actor–Partner Interdependent Model Test

**DOI:** 10.3390/ijerph18020487

**Published:** 2021-01-09

**Authors:** Miyoung Lee, Yeon-Suk Kim, Mi-Kyoung Lee

**Affiliations:** College of Nursing, Eulji University, 77, Gyeryong-ro, 771 beon-gil, Jung-gu, Daejeon 34824, Korea; mylee3730@eulji.ac.kr (M.L.); rladutn88@naver.com (Y.-S.K.)

**Keywords:** prenatal depression, actor–partner independent model test, marital intimacy

## Abstract

Prenatal depression is an important factor in predicting postpartum depression. Most studies have assessed factors affecting prenatal depression by focusing on pregnant wives. However, the emotional and psychological aspects of both expectant parents need to be considered. Therefore, the purpose of this study was to examine the effect of spouse-related stress in expectant couples on prenatal depression and investigate the mediating effects of marital intimacy on this relationship. A total of 120 expectant couples from two cities in Korea at more than 15 weeks of completed pregnancy participated in the study. Using a structured questionnaire, we assessed the general characteristics of the participants, spouse-related stress, prenatal depression, and marital intimacy. The results revealed that four actor effects and one partner effect were significant. Marital intimacy and prenatal depression among expectant parents were affected by spouse-related stress. Moreover, spouse-related stress in the husbands completely mediated marital intimacy in pregnant wives, demonstrating partner effects on prenatal depression in pregnant wives. Therefore, it was observed that paternal factors affect prenatal depression in pregnant wives. This warrants the inclusion of husbands in marital interventions and strategies to improve marital intimacy in pregnant wives.

## 1. Introduction

### 1.1. Background

Many pregnant wives experience physical, mental, and social changes, besides stress and depression, during pregnancy. In pregnant wives, depression may develop before and after childbirth. However, the vast majority of studies on depression in pregnant wives have focused more on postpartum depression than on prenatal depression. The prevalence of prenatal depression in pregnant wives has varied across studies. Approximately 10–35% of pregnant wives show symptoms of depression, and about 7% of pregnant wives experience depression after childbirth [1,2,3,4]. Pregnancy is also a significant stage in men’s lives as they prepare for fatherhood, and, like pregnant wives, men are known to experience prenatal depression [5,6]. A recent meta-analytic study found the prevalence rate of paternal prenatal depression to be 9.76% throughout the pregnancy period, although there were slight differences in the prevalence rate according to the stage of pregnancy [7]. This rate is slightly lower than that of prenatal depression in pregnant wives, indicating that paternal prenatal depression should not be overlooked.

Prenatal depression is one of the factors affecting postpartum depression; it is also a strong predictor of postpartum depression [8,9]. Depression in pregnant couples can act as a mediating factor in both partners, such that severe prenatal depression or depression in one spouse can increase their partner’s prenatal depression [10,11,12]. Factors affecting prenatal depression can be broadly divided into demographic, obstetric, and sociopsychological factors, wherein both women and men show common sociopsychological features. More specifically, higher stress during pregnancy in both men and women [6,13,14] and lower qualitative satisfaction with the marital relationship have been shown to cause higher prenatal depression [6,15,16].

During pregnancy, couples experience stress caused by various pregnancy-related concerns such as anxiety about the delivery, worries about the health of the fetus, and spousal changes in emotion [5,6,17]. A meta-analytic study found that difficulties faced in marital relationships greatly influence prenatal depression among both men and women [15,16], indicating that marital stress has significant negative effects on prenatal depression.

Intimacy is the concept of establishing a deep interpersonal relationship. It is an essential emotion for couples, the smallest unit of a family [18]. Lack of intimacy in a marriage causes sociopsychological maladjustment, depression, emotional disorders, and postpartum depression; moreover, the degree of marital intimacy may vary depending on the level of stress in the spousal relationship [19,20,21]. In particular, low marital intimacy causes emotional confusion and depression in pregnant wives who are already undergoing physical, mental, and social changes. Thus, husbands need to help their pregnant wives adapt to the various changes they are experiencing [22,23,24]. In other words, high marital intimacy is a positive factor that can promote emotional stability in both partners, acting as a buffer against stress in their relationship. Therefore, it is necessary to clarify the role of marital intimacy in the relationship between prenatal depression and spouse-related stress.

Although stress and relationship quality—as perceived by pregnant wives and their husbands during pregnancy and childbirth—are major factors affecting depression in both partners, many studies have mainly focused on pregnant wives. This has led to difficulties in establishing an effective prenatal depression relief intervention. A husband and wife are in a mutually complementary relationship that involves sharing and solving everyday problems. Accordingly, the emotional and psychological aspects of a pregnant couple should not be studied separately. Therefore, in this study, the actor–partner interdependent model (APIM) [25] was applied to couples in their second trimester to assess factors affecting prenatal depression besides factors of interaction between partners. In particular, the purpose of this study was to present a valid basis for developing a program to reduce prenatal depression by assessing the mediating effects of marital intimacy on the relationship between spouse-related stress and prenatal depression in expectant parents.

### 1.2. Objectives

This survey study assessed the relationship between spouse-related stress and prenatal depression in expectant parents. Furthermore, the mediating effects of marital intimacy on this relationship were investigated. The specific aims of this study were as follows:

1. To assess the level of spouse-related stress, marital intimacy, and prenatal depression among expectant parents.
2. To assess the correlation between spouse-related stress, marital intimacy, and prenatal depression among expectant parents.
3. To assess the actor–partner interdependent effects of spouse-related stress and marital intimacy on the prenatal depression in expectant parents.
4. To assess the mediating effects of marital intimacy on the relationship between spouse-related stress and prenatal depression in expectant parents.

## 2. Methods

### 2.1. Study Design

This was a cross-sectional survey study using APIM analysis to assess the effects of spouse-related stress and marital intimacy on prenatal depression in expectant parents. The APIM utilizes one-to-one data such as couples for analysis, and it is useful in identifying relationships [25].

### 2.2. Participants

After obtaining approval from the Institute Review Board of Eulji University (EU19-05), recruitment announcements for study participation were made in women’s hospitals and public health centers located in the S and D regions of Korea to recruit research participants. The inclusion criteria for couples were that the wives must have been pregnant for more than 15 weeks, neither partner was diagnosed with mental health problems, and both partners agreed to voluntarily participate in the study after understanding its purpose. The stage of pregnancy, over 15 weeks, was chosen as this is considered a relatively stable period at the beginning of the second trimester. The subjects who agreed to participate in the study completed a questionnaire following its explanation by the research assistant at the recruitment announcement agency. The questionnaire was self-reported, and the time required to complete the questionnaire was approximately 15 min. Data were collected from 6 February to 2 July 2019. A total of 125 pairs of questionnaires were distributed, and 123 pairs were received. Among the collected questionnaires, we analyzed data from 120 pairs (110 pregnant wives and 110 husbands), excluding three pairs of questionnaires answered by only one partner or with missing responses.

It was necessary to determine the sample size in the structural equation model in consideration of the complexity of the model (it was desirable to use a sample size 10 times greater than the number of free parameters) [26]. As a result, 120 expectant couples were included in the study, which satisfied the condition for the minimum sample size.

### 2.3. Measurement

#### 2.3.1. Spouse-Related Stress

This study used items on spouse-related areas from a pregnancy-related-stress measurement tool developed by Ahn [27] and evaluated by Lee and Seo [28] for reliability and validity. Each item was scored on a 5-point Likert scale. A score of 1 indicated “I am not worried at all,” and a score of 5 indicated “I am not comfortable at all” and “I am constantly worried.” Higher scores indicated higher spouse-related stress. In the study by Lee and Seo [28], Cronbach’s α, indicating the reliability of the results of spouse-related stress, was found to be 0.79. In this study, Cronbach’s α for spouse-related stress was 0.89 for pregnant wives and 0.83 for husbands.

#### 2.3.2. Marital Intimacy

This study used the intimacy subdomain of the Korean marriage satisfaction scale developed by Jeong [29] to measure marital intimacy. It was confirmed in the original paper that it is acceptable to use subdomains as individual scales depending on the needs of the researcher. The intimacy subdomain consisted of items on respect, expression of affection, free time spent together, and caring for one’s spouse. Each item was scored on a 4-point Likert scale, with a score of 1 indicating “not at all” and a score of 4 indicating “very much.” Higher scores indicated higher marital intimacy. Cronbach’s α for each sub-domain of the scale was in the range of 0.81–0.93. In this study, Cronbach’s α for marital intimacy was 0.96 for pregnant wives and 0.95 for husbands.

#### 2.3.3. Prenatal Depression

In this study, the modified Edinburgh Postnatal Depression Scale (EPDS), originally developed by Cox, Holden, and Sagovsky [30], was used to evaluate depression during pregnancy. The tool was originally developed to screen for postpartum depression; however, previous studies have demonstrated the validity of the tool to measure depression during pregnancy, as well [31,32]. The scale comprises 10 items, assessing depression, anxiety, and suicidal thoughts during the preceding week. The items are evaluated on a 4-point scale. Possible scores range from 0 to 30 points, with higher scores indicating more severe prenatal depression. In this study, Cronbach’s α for prenatal depression was 0.88 for pregnant wives and 0.71 for husbands.

### 2.4. Statistical Methods

The collected data were analyzed using SPSS 25.0 (IBM, Armonk, NY, USA) and Amos 25.0 (IBM, Armonk, NY, USA). The general characteristics of the study participants were analyzed using means, standard deviations, and percentages. Individual measurement variables were analyzed using descriptive statistics, and skewness and kurtosis were assessed to confirm the normality of the data. Cronbach’s α was calculated to assess the reliability of the measurement tools. The correlations between spouse-related stress, marital intimacy, and prenatal depression in the study participants were assessed using Pearson’s correlation coefficient. Following this, the measurement model was evaluated using construct reliability (CR) and average variance extracted (AVE). Finally, the study model was verified using chi-square (χ^2^), root mean square error of approximation (RMSEA), standardized root mean square residual (SRMR), comparative fit index (CFI), and Tucker–Lewis index (TLI).

## 3. Results

### 3.1. General Characteristics

The mean age of pregnant wives and their husbands in the study was 33 and 35 years, respectively. Regarding the educational level of the participants, most of the pregnant wives (70.8%) and their husbands (65.8%) had graduated from college. Moreover, most of the pregnant wives (53.3%) and their husbands (59.2%) were atheists. Regarding the degree of depression experienced in daily life, 57.5% of the wives answered that they were “not depressed,” 33.3% stated that they were “sometimes depressed,” and 9.2% said that they were “depressed.” Among the husbands, 70.8% responded that they were “not depressed,” 22.5% indicated that they were “sometimes depressed,” and 6.7% answered that they were “depressed.” The percentage of pregnant wives and husbands satisfied with their marriage was 92.5% and 97.5%, respectively (Table 1).

### 3.2. Descriptive Statistics and Validity Verification of Measurement Variables

Mean stress (pregnant wives = 2.57, husbands = 2.31), marital intimacy (pregnant wives = 3.07, husbands = 3.10), and depression (pregnant wives = 0.80, husbands = 0.55) were assessed (Table 2). In the univariate test for all measured variables, the skewness and kurtosis did not exceed the absolute values of 2 and 4, respectively, satisfying the condition for normality of the data [33] (Table 2). Regarding the confirmatory factor analysis (CFA) results, construct validity of AVE above 0.5 and CR above 0.7 were assessed. The AVE and CR of the latent variables were 0.61–0.89 and 0.72–0.94, respectively, demonstrating that the measurement variables sufficiently represented the six latent variables (Table 3). The correlation coefficient was −0.02~0.58, and stress was found to correlate negatively and positively with marital intimacy and depression, respectively (Table 3). In addition, marital intimacy was found to correlate negatively with depression.

### 3.3. Test of Measurement Invariance

The test of measurement invariance between the base model and the constrained model is considered significant if the result is higher than the χ^2^ difference for a given degree of freedom at a significant probability of 0.05 [33]. In the current study, this referred to different perceptions of the construct between the couples. In the basic model and constrained model, the χ^2^ difference was 6.08 (df = 6) (Table 4), which was lower than the χ^2^ difference of 12.59 for six degrees of freedom, indicating a nonsignificant result. In other words, it was observed that the expectant parents had the same perception of the measurement tools. This finding was meaningful for APIM analysis; thus, verification of the model was conducted.

### 3.4. Research Model Verification

**Figure 1:**
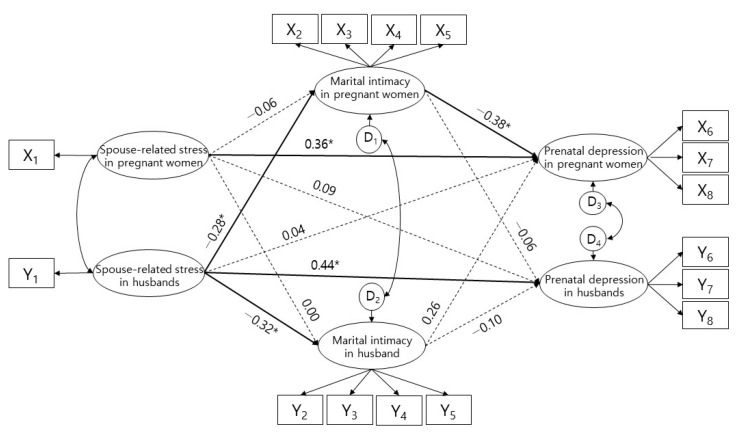
Results of hypothesis model verification. X1 = Spouse-related stress in pregnant women; X2 = Respect; X3 = Spending free time together; X4 = Caring for spouse; X5 = Expression of affection; X6 = Depression; X7 = Anxiety; X8= Suicidal thoughts; Y1 = Spouse-related stress in husbands; Y2 = Respect; Y3 = Spending free time together; Y4 = Caring for spouse; Y5 = Expression of affection; Y6 = Depression; Y7 = Anxiety; Y8 = Suicidal thoughts. * *p* < 0.05.

When the fitness of the model was evaluated, the fitness index was χ^2^/df = 1.30, SRMR = 0.07, CFI = 0.95, TLI = 0.93, and RMSEA = 0.05. The χ^2^/df, SRMR, and RMSEA were less than 3, 0.08, and 0.08, respectively. CFI and TLI were both higher than 0.90, suggesting an acceptable range of fit of the model. In the research model, a total of five paths were adopted as four actor effects, and one partner effect was found to be significant (Figure 1). Marital intimacy in the pregnant wives had direct partner effects on spouse-related stress in the husbands (β = −0.28, *p* = 0.031) with an explanatory power of 8.2%. In addition, spouse-related stress in the husbands had partner effects on prenatal depression in pregnant wives through complete mediation of marital intimacy in pregnant wives. Marital intimacy in the husbands had direct actor effects on spouse-related stress (β = −0.32, *p* = 0.010) in husbands with an explanatory power of 10.1%. Depression in pregnant wives had direct actor effects on spouse-related stress (β = 0.36, *p* = 0.010) and marital intimacy in pregnant wives (β = −0.38, *p* = 0.030) with an explanatory power of 23.8%. Depression in the husbands had direct actor effects on spouse-related stress (β = 0.44, *p* = 0.020) with an explanatory power of 26.3% (Table 5).

## 4. Discussion

Pregnancy is a joyful event in women’s lives; however, it is also a stressful event accompanied by physical changes and hormone-induced emotional changes. Previous studies on factors that influence depression have investigated pregnant women and expectant fathers separately, that is, studies have not involved couples. Therefore, in order to supplement the existing research, this study was conducted to examine the effects that partners may have on each other through analyzing couple data. Furthermore, this study attempted to assess the effects of spouse-related stress during pregnancy on prenatal depression by measuring marital intimacy through an analysis of actor and partner effects. The results of the study revealed many important implications.

First, the assessment of the actor and partner effects of spouse-related stress on prenatal depression showed that pregnant wives and their husbands had actor effects of increasing their own prenatal depression as spouse-related stress increased; no partner effects were observed in this regard. These findings were consistent with the results of previous studies [13,14], which have shown that stress acts as a major factor affecting prenatal depression. However, these results were also different from other studies [6], which found that stress experienced by one partner was related to depression suffered by both partners. Among the various factors influencing prenatal depression, there have been many studies on family and marital relationships. This study adds to the aforementioned literature by suggesting that spouse-related stress is a factor affecting prenatal depression. The stress measurement tools employed in this study consisted of items to assess stress resulting from difficulties in caring for one’s spouse due to pregnancy, including marital sexual activity. Therefore, educating couples on the changes and adaptations expected during pregnancy is necessary to alleviate prenatal depression. It is further suggested that information on maintaining a healthy sex life during pregnancy be included in this educational initiative.

Second, the assessment of the actor and partner effects of marital intimacy on prenatal depression showed that only actor effects were observed in the pregnant wives. There were no partner effects in either expectant parent. These results are consistent with the findings of previous studies showing that higher marital intimacy perceived by pregnant wives leads to less prenatal depression [34] and that the strength of a wife’s relationship with her husband, especially concerning marital intimacy, is closely related to her happiness when pregnant [35]. Marital intimacy is a process of emotional exchange that goes beyond simply maintaining a satisfactory relationship. It indicates a feeling of well-being experienced by partners in intellectual, physical, and emotional domains, and it plays a key role in sustaining family relationships [29]. Therefore, active communication between partners is fundamental to help pregnant women feel respected and loved, and experience a positive self-being during pregnancy. Such efforts will ultimately help to reduce prenatal depression.

Third, spouse-related stress in pregnant wives did not demonstrate actor or partner effects on marital intimacy. This finding contradicts the results of a previous study [36] reporting that the relationship between wives and their husbands acts as a buffer against stress during pregnancy. As described earlier, the current study did not reflect the various stress factors perceived by pregnant wives, resulting in the differences between these results and those of previous studies. Therefore, future studies on the relationship between spouse-related stress and marital intimacy in pregnant wives are necessary. In contrast to this study’s results regarding pregnant wives, for whom spouse-related stress had neither actor nor partner effects on marital intimacy, spouse-related stress in the husbands demonstrated negative actor and partner effects, suggesting that active management of this stress is necessary. This finding supports a previous qualitative study [5], which showed that husbands can experience various forms of stress during a partner’s pregnancy and that husbands perceive changes in their relationship with their wives during pregnancy, including experiencing less spousal intimacy. It has also been observed that emotional changes in the husbands, resulting from pregnancy, are as substantial as those of pregnant wives. Accordingly, a permissive social atmosphere acknowledging these emotional changes and providing support for the management of stress in husbands is required.

Finally, the effects of spouse-related stress on prenatal depression in consideration of marital intimacy were assessed in this study. In pregnant wives, spouse-related stress during pregnancy showed neither actor nor partner effects on marital intimacy. However, the opposite trend was observed in the husbands. It is noteworthy that spouse-related stress during pregnancy in husbands may aggravate prenatal depression by completely mediating the marital intimacy of pregnant wives. In other words, spouse-related stress in husbands can have positive or negative effects on prenatal depression in pregnant wives depending on the wives’ perceived level of marital intimacy. The findings of this study demonstrate the importance of interventions to improve the marital intimacy of pregnant wives. Such interventions need to be included in the development of future programs to help alleviate prenatal depression in pregnant wives.

Many studies have conducted APIM analyses to assess the dynamic interactions between middle-aged or elderly couples. However, this study is a novel attempt at assessing actor and partner effects on prenatal depression, especially among pregnant couples. Furthermore, this study showed that prenatal depression can be affected and experienced by both expectant parents. Thus, this study is significant in that it presents the basis for practical educational content that can be used in future prenatal depression intervention programs.

This study is limited in several respects. First, this study involved only 120 expectant couples residing in two cities in Korea, which limits the generalizability of its results. Second, due to the lack of previous studies implicating spouse-related stress as a factor affecting prenatal depression, a direct comparison between this study and previous studies’ results could not be made. In this regard, further studies on the relationship between spouse-related stress and prenatal depression are suggested. Additionally, as intimacy between partners can be affected by pregnancy, identifying changes by measuring levels of intimacy before, during, and after pregnancy is recommended. Third, in follow-up studies, it would be necessary to assess the validity of models by comparing and analyzing the paths between healthy pregnant wives and those suffering from prenatal depression.

## 5. Conclusions

This study utilized the APIM to examine the actor and partner effects of spouse-related stress on prenatal depression in pregnant wives and their husbands by considering marital intimacy during pregnancy. It was observed that the spouse-related stress and marital intimacy of pregnant wives had actor effects on prenatal depression. In the husbands, only spouse-related stress showed actor effects on prenatal depression. Furthermore, it was observed that spouse-related stress in the husbands affected prenatal depression in pregnant wives through the complete mediation of marital intimacy perceived by the wives. As a result, both maternal and paternal factors affected prenatal depression in pregnant wives, suggesting that husbands should also be considered in planning interventions to reduce prenatal depression. Besides, this study provided basic data to support the inclusion of strategies to improve the marital intimacy of pregnant wives in intervention programs.

## Figures and Tables

**Table 1:** General characteristics of the participants.

Variables	Categories	N (%)			
		Pregnant Wives (120)		Husbands (120)	
Age (years)		32.54 (3.83)		34.73 (3.90)	
Education	High school	13	(10.8)	14	(11.7)
	College	85	(70.8)	79	(65.8)
	Graduate	22	(18.3)	27	(22.5)
Religion	Yes	56	(46.7)	49	(40.8)
	No	64	(53.3)	71	(59.2)
	Depressed	11	(9.2)	8	(6.7)
Depression	Sometimes depressed	40	(33.3)	27	(22.5)
	Not depressed	69	(57.5)	85	(70.8)
Marital satisfaction	Satisfied	111	(92.5)	117	(97.5)
	Dissatisfied	9	(7.5)	3	(2.5)

**Table 2:** Descriptive statistics of variables.

Variables	Range	Pregnant Wives (n = 120)			Husbands (n = 120)		
		M ± SD	Skewness	Kurtosis	M ± SD	Skewness	Kurtosis
Spouse-related stress	0–5	1.97 ± 0.70	0.56	−0.07	1.74 ± 0.72	0.95	0.88
Marital intimacy	0–4	3.07 ± 0.60	−0.47	−0.01	3.10 ± 0.54	−0.65	1.25
Respect		3.02 ± 0.66	−0.40	−0.14	2.91 ± 0.65	−0.27	−0.08
Spending free time together		3.27 ± 0.63	−0.94	1.61	3.26 ± 0.60	−0.96	1.70
Caring for spouse		3.16 ± 0.72	−0.83	0.27	3.19 ± 0.60	−0.57	0.76
Expression of affection		2.92 ± 0.74	−0.32	−0.38	2.96 ± 0.76	−0.54	0.07
Depression	0–3	0.80 ± 0.52	1.08	1.58	0.55 ± 0.40	0.76	0.08
Depression		0.55 ± 0.63	1.35	1.93	0.25 ± 0.42	1.93	3.46
Anxiety		0.65 ± 0.52	1.19	1.21	0.56 ± 0.49	0.88	0.62
Suicidal thoughts		1.21 ± 0.67	0.29	−0.52	0.86 ± 0.65	0.40	−0.68

Note. M = mean; SD = standard deviation.

**Table 3:** Number of correlations and validity of variables (N = 240).

Variables	Spouse-Related Stress in Pregnant Women	Marital Intimacy in Pregnant Women	Prenatal Depression in Pregnant Women	Spouse-Related Stress in Husbands	Martial Intimacy in Husbands	Prenatal Depression in Husbands
Spouse-related stress in pregnant women	1					
Marital intimacy in pregnant women	−0.08	1				
Prenatal depression in pregnant women	0.30 **	−0.22 *	1			
Spouse-related stress in husbands	0.14	−0.10	0.02	1		
Martial intimacy in husbands	0.01	0.58 **	−0.02	−0.12	1	
Prenatal depression in husbands	−0.02	−0.23 *	0.19 *	0.20 *	−0.29 **	1
CR	0.82	0.94	0.91	0.78	0.94	0.90
AVE	0.61	0.89	0.77	0.50	0.79	0.74

Note. AVE = average variance extracted; CR = construct reliability. * *p* < 0.05, ** *p* < 0.01.

**Table 4:** Measurement invariance test.

Index	Base (A)	Constrained (B)
$x^{2}$	166.26	172.35
*df*	111 (*p* = 0.001)	117 (*p* = 0.001)
$x^{2}$/df	1.50	1.47
SRMR	0.07	0.07
CFI	0.94	0.94
TLI	0.92	0.92
RMSEA	0.07	0.06
$∆x^{2}$/*p*	6.08/0.414	

Note. SRMR = standardized root mean square residual; CFI = comparative fit index; TLI = Tucker–Lewis index; RMSEA = root mean square error of approximation.

**Table 5:** Model verification.

Endogenous Variable	Exogenous Variable	SE	CR (*p*)	SMC	Direct Effect	Indirect Effect	Total Effect
Martial intimacy in pregnant women	Spouse-related stress in pregnant women	0.09	−0.60 (0.548)	0.082	−0.06 (0.608)		−0.06 (0.608)
	Spouse-related stress in husbands	0.13	−2.53 (0.011)		−0.28 (0.031)		−0.28 (0.031)
Marital intimacy in husbands	Spouse-related stress in pregnant women	0.07	0.03 (0.975)	0.101	0.00 (0.990)		0.00 (0.990)
	Spouse-related stress in husbands	0.12	−2.79 (0.005)		−0.32 (0.010)		−0.32 (0.010)
Prenatal depression in pregnant women	Spouse-related stress in pregnant women	0.08	3.24 (0.001)	0.238	0.36 (0.010)	0.02 (0.591)	0.29 (0.010)
	Spouse-related stress in husbands	0.12	0.32 (0.749)		0.04 (0.858)	0.02 (0.549)	0.06 (0.705)
	Marital intimacy in pregnant women	0.12	−2.71 (0.007)		−0.38 (0.030)		−0.38 (0.030)
	Marital intimacy in husbands	0.14	1.83 (0.067)		0.26 (0.112)		0.26 (0.112)
Prenatal depression in husbands	Spouse-related stress in pregnant women	0.05	0.80 (0.427)	0.263	0.09 (0.378)	0.00 (0.903)	0.04 (0.384)
	Spouse-related stress in husbands	0.09	3.06 (0.002)		0.44 (0.020)	0.03 (0.410)	0.30 (0.010)
	Marital intimacy in pregnant women	0.08	−0.41 (0.681)		−0.06 (0.679)		−0.06 (0.679)
	Marital intimacy in husbands	0.09	−0.65 (0.517)		−0.10 (0.712)		−0.10 (0.712)

Note. SE = standardized error; CR = critical ratio; SMC = squared multiple correlations.
